# Carbon emissions associated with antenatal testing for hepatitis B prophylaxis eligibility, the Gambia

**DOI:** 10.2471/BLT.25.294507

**Published:** 2026-03-04

**Authors:** Alassane Ndiaye, Florian Motyl, Sainabou Drammeh, Bakary Dibba, Alexandra Famiglietti, Maya Whittaker, Kévin Jean, Maud Lemoine, Sylvie Boyer, Dramane Kania, Alice Nanelin Guingané, Naofumi Hashimoto, Yasuhito Tanaka, Wataru Sugiura, Kris Murray, Christopher Vandi, Umberto D’Alessandro, Florence Guivel-Benhassine, Hélène Da Conceicao, Guillaume Pakula, Gibril Ndow, Yusuke Shimakawa

**Affiliations:** a Institut Pasteur, Université Paris Cité, Unité d’Épidémiologie des Maladies Émergentes, 25–28 rue du Dr Roux, 75015, Paris, France.; b Projet Celsius, Marseille, France.; c Medical Research Council Unit The Gambia at the London School of Hygiene and Tropical Medicine, Fajara, the Gambia.; d Faculty of Biology, Medicine and Health, University of Manchester, Manchester, England.; e Institut de Biologie de l’École Normale Supérieure, CNRS, Inserm, Université Paris Science & Lettres, Paris, France.; f Department of Metabolism, Digestion and Reproduction, Imperial College London, London, England.; g Sciences économiques et sociales de la santé & traitement de l’information médicale, Aix-Marseille Université, Marseille, France.; h Center Muraz, Institut National de Santé Publique, Bobo-Dioulasso, Burkina Faso.; i Hepato-Gastroenterology Department, Bogodogo University Hospital Center, Ouagadougou, Burkina Faso.; j Bureau of International Health Cooperation, Japan Institute for Health Security, Tokyo, Japan.; k Department of Gastroenterology and Hepatology, Kumamoto University, Kumamoto, Japan.; l Center for Clinical Sciences, Japan Institute for Health Security, Tokyo, Japan.; m Virus and Immunity Unit, Institut Pasteur, Paris, France.; n Responsabilité Sociétale des Entreprises, Institut Pasteur, Paris, France.

## Abstract

**Objective:**

To estimate the carbon footprint of three diagnostic strategies to identify pregnant women eligible for antiviral prophylaxis to prevent hepatitis B vertical transmission in the Gambia.

**Methods:**

In 2024, we conducted a life cycle assessment of a point-of-care polymerase chain reaction (PCR) test using plasma, and a rapid diagnostic test for hepatitis B core-related antigen (HBcrAg) using plasma and capillary blood across three hospitals (rural, suburban and urban) and a suburban health centre. We included all products and processes in each diagnostic strategy. The functional unit was an antenatal testing episode assessing eligibility for antiviral prophylaxis, beginning after positive hepatitis B surface antigen screening. We estimated carbon emissions in grams of carbon dioxide equivalent (g CO_2_e) ± uncertainty.

**Findings:**

Mean carbon emissions per strategy were significantly different between point-of-care PCR and the rapid diagnostic tests (*P*-value: 0.028): 1619.0 ± 200.6 g CO_2_e (PCR), 520.4 ± 59.1 g CO_2_e (plasma-based rapid diagnostic test) and 374.3 ± 50.4 g CO_2_e (capillary-based test). Higher emissions with the PCR test were mainly driven by its reliance on air conditioning (759.1 g CO_2_e compared with 125.2 g CO_2_e for plasma-based rapid diagnostic test and 24.3 g CO_2_e for capillary-based test); the test itself (290.0 g CO_2_e versus 129.0 g CO_2_e for rapid diagnostic tests); and PCR-specific requirements including diagnostic device (47.0 g CO_2_e) and additional patient travel to collect results (255.8 g CO_2_e).

**Conclusion:**

Our findings suggest that HBcrAg rapid diagnostic tests can reduce emissions substantially compared with point-of-care PCR. Our study demonstrates that life cycle assessments are feasible in resource-constrained settings and highlights the importance of integrating sustainability into hepatitis B diagnostic strategies.

## Résumé

**Objectif:**

Évaluer l’impact carbone de trois stratégies de diagnostic visant à identifier les femmes enceintes éligibles à la prophylaxie antivirale pour prévenir la transmission verticale de l’hépatite B en Gambie.

**Méthodes:**

En 2024, nous avons réalisé une analyse du cycle de vie d’un test de réaction en chaîne par polymérase (PCR) au point de service à l’aide de plasma, ainsi qu’un test de diagnostic rapide de l’antigène lié au core de l’hépatite B (HBcrAg) à l’aide de plasma et de sang capillaire dans trois hôpitaux (rural, suburbain et urbain) et un centre de santé suburbain. Nous avons inclus tous les produits et processus de chaque stratégie de diagnostic. L’unité fonctionnelle était un épisode de test prénatal évaluant l’éligibilité à la prophylaxie antivirale, qui commençait à l’issue d’un dépistage positif de l’antigène de surface de l’hépatite B. Nous avons estimé les émissions de carbone en grammes d’équivalent de dioxyde de carbone (g éq. CO_2_) ainsi qu’une plage d’incertitude.

**Résultats:**

Les émissions moyennes de carbone par stratégie étaient nettement différentes entre la PCR au point de service et les tests de diagnostic rapide (valeur *P*: 0,028): 1619,0 ± 200,6 g éq. CO_2_ (PCR), 520,4 ± 59,1 g éq. CO_2_ (test de diagnostic rapide à partir de plasma) et 374,3 ± 50,4 g éq. CO_2_ (test capillaire). Les émissions plus élevées de la PCR s’expliquent principalement par son recours à la climatisation (759,1 g éq. CO2 contre 125,2 g éq. CO_2_ pour le test de diagnostic rapide à partir de plasma et 24,3 g éq. CO_2_ pour le test capillaire), le test lui-même (290,0 g éq. CO_2_ contre 129,0 g éq. CO_2_ pour les tests de diagnostic rapide) et les exigences spécifiques à la PCR, notamment le dispositif de diagnostic (47,0 g éq. CO_2_) et le transport médical supplémentaire pour récupérer les résultats (255,8 g éq. CO_2_).

**Conclusion:**

Nos résultats suggèrent que les tests de diagnostic rapide de l’HBcrAg permettent de réduire considérablement l’impact carbone par rapport à la PCR au point de service. Notre étude démontre que les analyses du cycle de vie sont réalisables dans des contextes de ressources limitées et souligne l’importance d’intégrer la durabilité dans les stratégies de diagnostic de l’hépatite B.

## Resumen

**Objetivo:**

Estimar la huella de carbono de tres estrategias diagnósticas para identificar a las mujeres embarazadas elegibles para profilaxis antiviral con el fin de prevenir la transmisión vertical de la hepatitis B en Gambia.

**Métodos:**

En 2024, se realizó una evaluación del ciclo de vida de una prueba de reacción en cadena de la polimerasa (PCR) en el punto de atención utilizando plasma, y de una prueba diagnóstica rápida para el antígeno relacionado con el núcleo de la hepatitis B (HBcrAg) utilizando plasma y sangre capilar en tres hospitales (rural, suburbano y urbano) y un centro de salud suburbano. Se incluyeron todos los productos y procesos de cada estrategia diagnóstica. La unidad funcional fue un episodio de pruebas prenatales para evaluar la elegibilidad para profilaxis antiviral, iniciado tras un resultado positivo en el cribado del antígeno de superficie de la hepatitis B. Se estimaron las emisiones de carbono en gramos de dióxido de carbono equivalente (g CO₂ eq.) ± incertidumbre.

**Resultados:**

Las emisiones medias de carbono por estrategia fueron significativamente diferentes entre la PCR en el punto de atención y las pruebas diagnósticas rápidas (valor de *p*: 0,028): 1619,0 ± 200,6 g CO₂ eq. (PCR), 520,4 ± 59,1 g CO₂ eq. (prueba diagnóstica rápida basada en plasma) y 374,3 ± 50,4 g CO₂ eq. (prueba basada en sangre capilar). Las mayores emisiones asociadas a la prueba de PCR se debieron principalmente a su dependencia del aire acondicionado (759,1 g CO₂ eq. en comparación con 125,2 g CO₂ eq. para la prueba diagnóstica rápida basada en plasma y 24,3 g CO₂ eq. para la prueba basada en sangre capilar), a la propia prueba (290,0 g CO₂ eq. frente a 129,0 g CO₂ eq. para las pruebas diagnósticas rápidas) y a requisitos específicos de la PCR, incluido el dispositivo diagnóstico (47,0 g CO₂ eq.) y los desplazamientos adicionales de las pacientes para recoger los resultados (255,8 g CO₂ eq.).

**Conclusión:**

Los resultados sugieren que las pruebas diagnósticas rápidas del HBcrAg pueden reducir sustancialmente las emisiones en comparación con la PCR en el punto de atención. El estudio demuestra que las evaluaciones del ciclo de vida son viables en entornos con recursos limitados y destaca la importancia de integrar la sostenibilidad en las estrategias diagnósticas de la hepatitis B.

## ملخص

الغرض تقدير البصمة الكربونية لثلاث استراتيجيات تشخيصية لتحديد النساء الحوامل المؤهلات لتلقي العلاج الوقائي المضاد للفيروسات، للوقاية من انتقال التهاب الكبد ب بشكل رأسي في غامبيا.

الطريقة في عام 2024، قمنا بإجراء تقييم لدورة حياة اختبار تفاعل البوليميراز المتسلسل (PCR) الذي يتم في نقاط الرعاية باستخدام البلازما، واختبار تشخيصي سريع للمستضد الرئيسي المرتبط بالتهاب الكبد ب (HBcrAg)، وذلك باستخدام البلازما ودم الشعيرات الدموية في ثلاثة مستشفيات (ريفية، وفي الضواحي، وحضرية) ومركز صحي في الضواحي. قمنا بتضمين جميع المنتجات والعمليات في كل استراتيجية تشخيصية. كانت الوحدة الوظيفية هي فحص ما قبل الولادة لتقييم أهلية النساء الحوامل لتلقي العلاج الوقائي المضاد للفيروسات، والذي يبدأ بعد نتيجة إيجابية لفحص مستضد سطح التهاب الكبد ب. قمنا بتقدير انبعاثات الكربون بالجرام من مكافئ ثاني أكسيد الكربون (جرام CO2e) ± هامش الخطأ.

النتائج كان متوسط انبعاثات الكربون لكل استراتيجية مختلفًا بشكل كبير بين اختبار PCR عند نقطة الرعاية واختبارات التشخيص السريع (نسبة الاحتمال: 0.028): 1619.0 ± 200.6 جرام CO2e (PCR)، 520.4 ± 59.1 جم CO2e (اختبار التشخيص السريع القائم على البلازما) و374.3 ± 50.4 جرام CO2e (الاختبار القائم على الشعيرات الدموية). تعود الانبعاثات الأعلى لاختبار تفاعل البوليميراز المتسلسل (PCR) بشكل رئيسي إلى اعتماده على تكييف الهواء (759.1 جرام من CO2e مقارنةً بـ 125.2 جرام من CO2e لاختبار التشخيص السريع القائم على البلازما، و24.3 جرام من CO2e لاختبار التشخيص القائم على الشعيرات الدموية)، والاختبار نفسه (290.0 جرام من CO2e مقابل 129.0 جرام من CO2e لاختبارات التشخيص السريع)، ومتطلبات اختبار PCR الخاصة، بما في ذلك جهاز التشخيص (47.0 جرام من CO2e) وانتقال المريض الإضافي لجمع النتائج (255.8 جرام من CO2e).

الاستنتاج تشير النتائج لدينا إلى أن اختبارات التشخيص السريع لمستضد HBcrAg يمكن أن تقلل من الانبعاثات بشكل كبير مقارنةً باختبار PCR الذي يُجرى في نقاط الرعاية. تُظهر الدراسة لدينا أن تقييمات دورة الحياة ممكنة في البيئات ذات الموارد المحدودة، وتُبرز أهمية دمج الاستدامة في استراتيجيات تشخيص التهاب الكبد ب.

## 摘要

**目的:**

旨在估算冈比亚为防止乙肝垂直传播而推出的三种诊断策略（用于确定具备接受抗病毒预防治疗资格的孕妇）的碳足迹。

**方法:**

我们于 2024 年在三所医院（分别位于农村地区、郊区和城区）和一个郊区卫生保健中心对以下两项检测进行了生命周期评估：以血浆为样本的即时聚合酶链反应 (PCR) 检测；以血浆和毛细血管血为样本的乙肝核心相关抗原 (HBcrAg) 快速诊断检测。我们将所有产品和流程都纳入了每种诊断策略。功能单位是在得知乙肝表面抗原筛查结果呈阳性后评估是否具备接受抗病毒预防治疗资格的产前检测阶段。我们估算碳排放量时采用克二氧化碳当量 (g CO_2_e) 为单位，并考虑了不确定性区间。

**结果:**

每种策略的平均碳排放量在即时 PCR 和快速诊断检测之间存在明显差异（P 值：0.028）：1619.0 ± 200.6 g CO_2_e (PCR)；520.4 ± 59.1 g CO_2_e（基于血浆的快速诊断检测）和 374.3 ± 50.4 g CO2e（基于毛细血管血的快速诊断检测）。PCR 检测的碳排放量较高的主要原因有三个：该项检测依赖于空调系统【759.1 g CO_2_e，相比之下，快速诊断检测分别为 125.2 g CO_2_e（基于血浆）和 24.3 g CO_2_e（基于毛细血管血）】；检测本身（290.0 g CO_2_e，相比之下，快速诊断检测为 129.0 g CO_2_e）；以及 PCR 相关特定要求【包括诊断设备 (47.0 g CO_2_e) 和患者为领取结果而另外安排的出行 (255.8 g CO_2_e)】 。

**结论:**

我们的结果表明，与即时 PCR 相比，HBcrAg 快速诊断检测能够明显降低碳排放量。我们的研究证明了可在资源有限的地区开展生命周期评估，并强调了将可持续发展纳入乙肝诊断策略的重要性。

## Резюме

**Цель:**

Оценить углеродный след трех диагностических стратегий для выявления беременных женщин, которым показана противовирусная профилактика с целью предотвращения вертикальной передачи гепатита B в Гамбии.

**Методы:**

В 2024 году авторы провели оценку жизненного цикла для ПЦР-теста (теста на основе полимеразной цепной реакции) по месту оказания медицинской помощи с использованием плазмы, а также для экспресс-теста на антиген, связанный с ядром вируса гепатита B (HBcrAg), с использованием плазмы и капиллярной крови в трех больницах (сельской, пригородной и городской) и одном пригородном медицинском центре. Были включены все продукты и процессы, входящие в каждую диагностическую стратегию. Функциональной единицей являлся один эпизод антенатального тестирования для оценки показаний к противовирусной профилактике, начинавшийся после положительного скрининга на поверхностный антиген гепатита B. Были оценены выбросы углерода в граммах эквивалента диоксида углерода (г CO_2_-экв) ± неопределенность.

**Результаты:**

Средние выбросы углерода для каждой стратегии значительно различались между ПЦР по месту оказания медицинской помощи и экспресс-тестами (P-значение: 0,028): 1619,0 ± 200,6 г CO_2_-экв (ПЦР), 520,4 ± 59,1 г CO_2_-экв (экспресс-тест на основе плазмы) и 374,3 ± 50,4 г CO_2_-экв (тест на основе капиллярной крови). Более высокие выбросы при использовании ПЦР-теста в основном были обусловлены его зависимостью от кондиционирования воздуха (759,1 г CO_2_-экв по сравнению с 125,2 г CO_2_-экв для экспресс-теста на основе плазмы и 24,3 г CO_2_-экв для теста на основе капиллярной крови), самим тестом (290,0 г CO_2_-экв против 129,0 г CO_2_-экв для экспресс-тестов), а также специфическими требованиями ПЦР, включая диагностический прибор (47,0 г CO_2_-экв), и дополнительными поездками пациентов для получения результатов (255,8 г CO_2_-экв).

**Вывод:**

Полученные результаты показывают, что экспресс-тесты на HBcrAg могут существенно сократить выбросы по сравнению с ПЦР в месте оказания медицинской помощи. Наше исследование демонстрирует, что оценка жизненного цикла осуществима в условиях ограниченных ресурсов и подчеркивает важность интеграции принципов устойчивости в стратегии диагностики гепатита B.

## Introduction

In 2024, the World Health Assembly recognized climate change as an urgent threat to global health.^1^ By intensifying extreme weather events, climate change worsens mental health and increases disease transmission and mortality.^2^^,^^3^ The World Health Organization (WHO) estimates that 250 000 additional deaths annually will occur between 2030 and 2050, mainly from malnutrition, malaria, diarrhoea and heat-related illnesses.^2^ These impacts are expected to disproportionately affect populations in low- and middle-income countries.^4^ Urgent action to reduce greenhouse gas emissions is vital to mitigate these effects.

Despite contributing an estimated 4.6% of global carbon emissions,^5^ the health-care sector’s environmental impact is often overlooked in health technology assessments, which are the main tool used by health systems to inform the adoption of health technologies or interventions and reimbursement decisions.^6^ While most research on health-care-related emissions has focused on hospitals,^7^ medical equipment,^8^^,^^9^ consumables,^10^ treatment,^11^ pharmaceuticals^12^ and hospital waste,^13^ diagnostics are underexplored. Few studies have compared the greenhouse gas emissions of diagnostic tools used for the same clinical indication.^14^^,^^15^

Diagnostic tools are essential for managing infectious diseases, particularly in low- and middle-income countries, where health systems face increasing clinical and environmental challenges such as high infectious disease burden, constrained health-care resources and infrastructure, and growing environmental pressures on health systems. Hepatitis B virus (HBV) infection, responsible for an estimated 1.1 million deaths in 2022, is the second leading infectious cause of death globally after tuberculosis.^16^ In many low- and middle-income countries, especially in sub-Saharan Africa, prevention of mother-to-child transmission (PMTCT) of HBV is central to achieving hepatitis B elimination.^16^ PMTCT involves antenatal screening for hepatitis B surface antigen (HBsAg); quantification of HBV deoxyribonucleic acid (DNA) to identify women eligible for antiviral prophylaxis; and administration of a hepatitis B birth-dose vaccine to all infants irrespective of maternal HBV status.^17^ While real-time polymerase chain reaction (PCR) is the reference method for HBV DNA quantification, its use is still limited in many low- and middle-income countries due to high costs, infrastructure needs and workforce shortages.^17^

The hepatitis B core-related antigen (HBcrAg) rapid diagnostic test (ESPLINE^TM^, Fujirebio, Japan) is a promising alternative to PCR-based testing. This lateral-flow assay can use capillary blood, requires no laboratory infrastructure or electricity, and has a sensitivity of 93.1% (95% confidence interval, CI: 90.5–95.2) and specificity of 94.3% (95% CI: 93.0–95.4) in identifying women eligible for antiviral prophylaxis.^18^ This test was recently CE-marked, that is, the product complies with all relevant European Union legislation, as a highly sensitive rapid test for the detection of hepatitis B e antigen (HBeAg; ESPLINE^TM^ HBeAg-hs). Furthermore, in a micro-costing study in Burkina Faso, the HBcrAg rapid diagnostic test reduced testing costs by up to 40.0% compared with PCR.^19^ However, the environmental impact of the test, an important consideration for sustainable health systems, has not been assessed.

In this study, we compared the carbon dioxide equivalent (CO_2_e) emissions of three diagnostic strategies for identifying HBsAg-positive pregnant women eligible for antiviral prophylaxis in the Gambia: (i) point-of-care PCR (Xpert® HBV Viral Load, Cepheid, United States of America) using plasma; (ii) the HBcrAg rapid diagnostic test using plasma; and (iii) the HBcrAg rapid diagnostic test using capillary blood. Using a process-based life cycle assessment, we aimed to provide context-specific carbon footprint data to guide HBV prevention strategies in low- and middle-income countries.

## Methods

### Study setting

The Gambia, a low-income country in West Africa, has a population of 2.7 million. This analysis was conducted within two research projects: the TRI-MOM (Triple Elimination Model of Mother-to-Child Transmission of HIV, Syphilis, and HBV) and its ancillary study, PROTECT-B (Performance and Feasibility of Hepatitis B Core-Related Antigen Rapid Test). Ethical approval was obtained from the Gambia Government/Medical Research Council Joint Ethics Committee (ref: 30302).

**Fig. 1:**
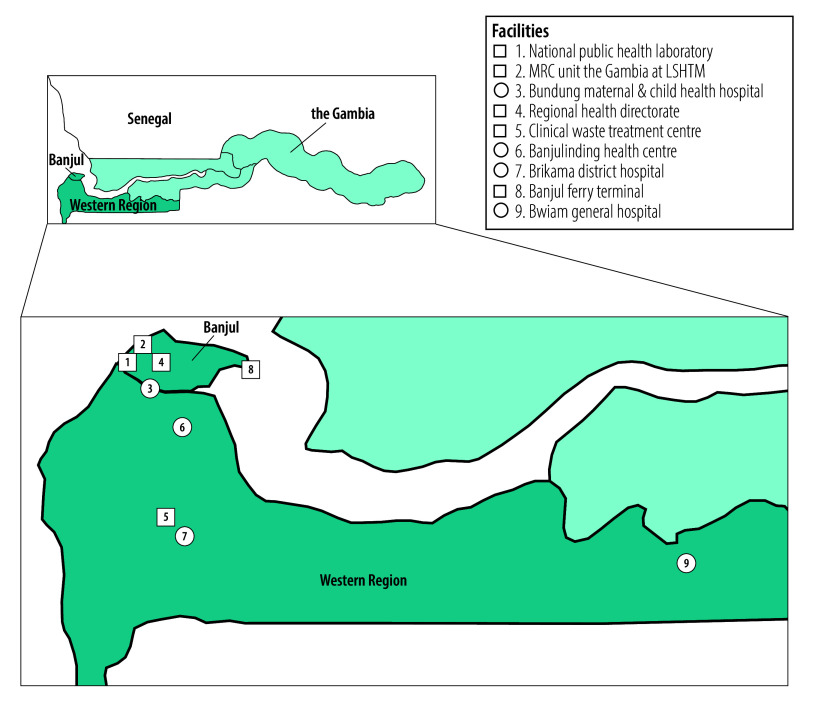
Map of the health-care facilities included in the study on carbon emissions associated with antenatal testing for hepatitis B prophylaxis eligibility, the Gambia, 2024

MRC: Medical Research Council Unit; LSHTM: London School of Hygiene and Tropical Medicine

Started in 2022, TRI-MOM evaluates the integration of hepatitis B PMTCT into routine antenatal care alongside human immunodeficiency virus (HIV) and syphilis screening in the Gambia and Burkina Faso. The project selected four health-care facilities in the Gambia’s Western Region to reflect different settings (Fig. 1): Bwiam general hospital (rural), Banjulinding health centre (suburban), Bundung maternal and child health hospital (urban) and Brikama district hospital (suburban). After informed consent, pregnant women were screened for HIV, syphilis and hepatitis B using two rapid diagnostic tests: Bioline HIV/syphilis duo and Determine HBsAg 2 (Abbott, Illinois, USA). At three facilities, HBsAg-positive women were tested using a point-of-care PCR for HBV DNA, and the women with viral loads ≥ 200 000 IU/mL received tenofovir disoproxil fumarate as antiviral prophylaxis. At Banjulinding, where PCR was unavailable, a treat-all approach was used in which all HBsAg-positive women received tenofovir disoproxil fumarate. Samples from HBsAg-positive women were systematically sent to the Medical Research Council Unit the Gambia laboratory for HBV DNA testing.

PROTECT-B, conducted between 2024 and 2025, was designed to validate HBcrAg rapid diagnostic test performance using venous plasma and finger-prick capillary blood, with a point-of-care PCR performed on venous plasma as the reference. The HBcrAg rapid diagnostic test was introduced into routine antenatal care and performed on-site in HBsAg-positive women. Although the results were not used to guide treatment, care pathway modelling assumed HBcrAg-positive women would receive same-day tenofovir disoproxil fumarate, consistent with a simplified test-and-treat approach.

### Scope and functional unit

We conducted a life cycle assessment following the Product Life Cycle Accounting and Reporting Standard,^20^ the Greenhouse Gas Accounting Sector Guidance for Pharmaceutical Products and Medical Devices^21^ and ISO 14044:2006.^22^ Greenhouse gas emissions were expressed as CO_2_e, the study’s main outcome.^20^ The functional unit was a single antenatal testing episode to determine eligibility for peripartum antiviral prophylaxis, beginning after a positive HBsAg screening test and ending with receipt of results from either the point-of-care PCR or rapid diagnostic test.

### System boundary

**Fig. 2:**
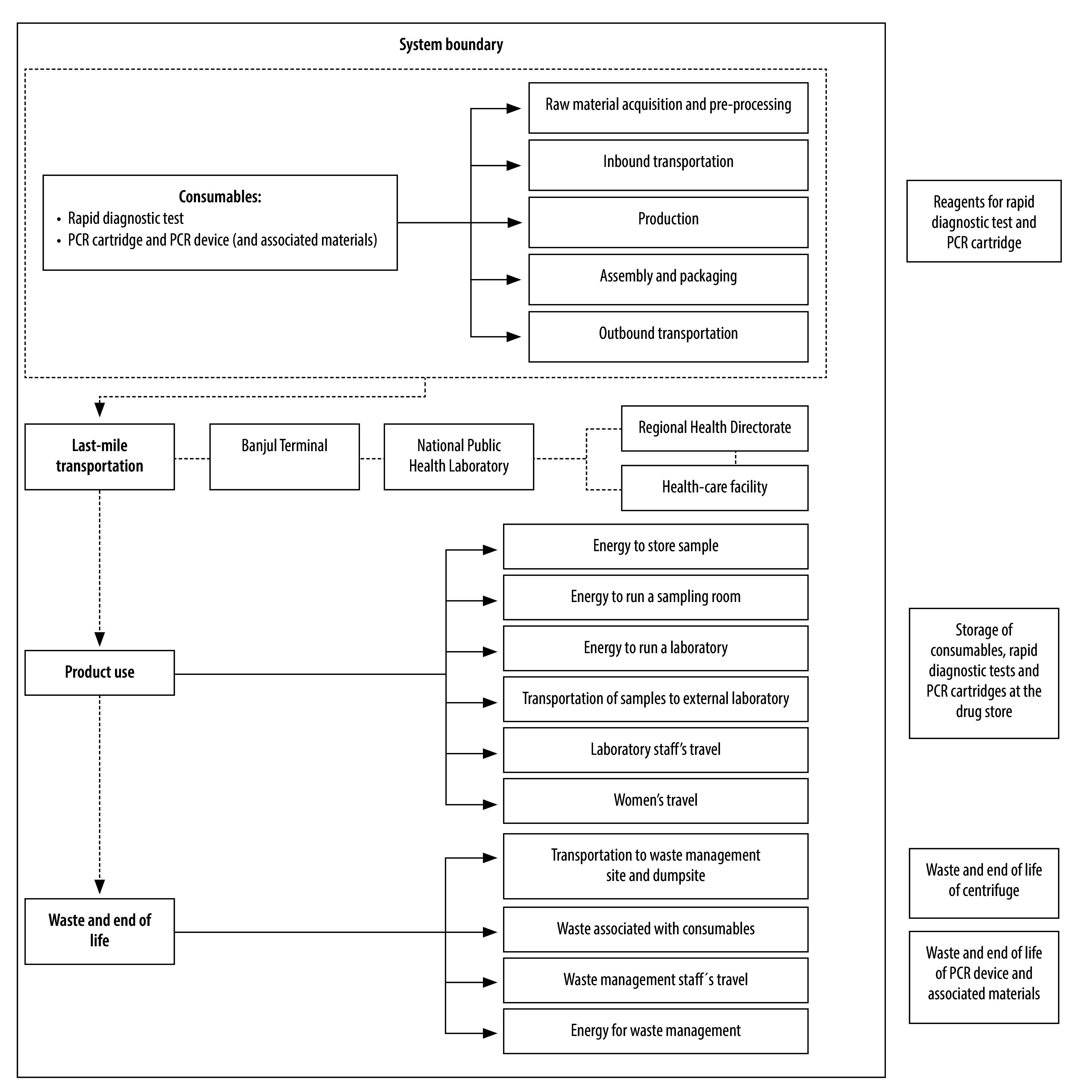
System boundary of life cycle assessment of diagnostic strategies

This life cycle assessment followed a cradle-to-grave approach, covering the following stages: 1: raw material acquisition and pre-processing; 2: inbound transportation; 3: production; 4: assembly and packaging; 5: outbound transportation; 6: last-mile transportation; 7: product use; and 8: end-of-life and waste management.^23^ We used both primary and secondary data. Between 1 July and 31 August 2024, we obtained field data from the Gambia for stages 6 to 8, and sourced data for stages 1 to 5 from a Unitaid assessment of the point-of-care PCR for *Mycobacterium tuberculosis* and rifampicin resistance (Xpert® MTB/RIF, Cepheid, USA) assay^23^ and the manufacturer of the HBcrAg rapid diagnostic test, both of which are consistent with the Greenhouse Gas Protocol.^20^ Fig. 2 shows the system boundary and included processes.

PCR: polymerase chain reaction.

Stage 1 included consumables and equipment for blood collection and testing. For the HBcrAg rapid diagnostic test, we excluded buffer solution emissions due to negligible weight (< 0.001 g). For point-of-care PCR, we omitted reagents, as data were unavailable for these components. Stage 2 considered transport of raw materials to manufacturers. Stages 3 and 4 covered production, assembly and packaging of test components and consumables, including gloves, swabs, pipettes, tubes and the PCR device. Stage 5 assumed sea freight from manufacturers to the Gambia. Stage 6 covered last-mile distribution from the Banjul Ferry Terminal to the National Public Health Laboratory, and onward to regional health directorates and health-care facilities. Stage 7 included electricity use for blood collection and testing (for example, lighting, fans, air conditioning, refrigerators, centrifuges, biosafety hoods and PCR equipment), transport to external laboratories when PCR was off-site (i.e. Banjulinding) and patient return travel if their test results were not available on the same day as their initial HBsAg screening results. We excluded storage emissions due to minimal space requirements. We included staff commuting and meals. Stage 8 covered medical waste transport and treatment (autoclaving, shredding or incineration), both on-site and off-site. We also included end-of-life disposal of the PCR device, but excluded peripheral electronics (e.g. laptops, scanners and centrifuges), assuming continued use beyond the operational life of the PCR device. We also considered waste management staff travel.

### Data collection

We collected activity data and emission factors for all components within the system boundary of each diagnostic strategy. Activity data included electricity use (in kWh), material weight (in kg), travel distances (in km) and time spent (in h). We collected primary data through direct observation and structured interviews, with informed consent, at the four study sites and through consultations with the health ministry and the Medical Research Council Unit the Gambia. Interviewees included health workers, laboratory staff, logistics staff, waste handlers and researchers. We pretested a structured questionnaire and refined after pilot testing at Medical Research Council Unit the Gambia. We compiled a detailed inventory of items and activities using process mapping and system boundary criteria.

For stage 6 (last-mile transport), we obtained vehicle types, routes and delivery frequencies from facility records; we extrapolated transport volumes from Medical Research Council Unit the Gambia data. For stage 7 (use phase), we documented and weighed all medical consumables (e.g. gloves, needles, pipettes and test kits) on-site with a Denver Instruments S-402® scale (Denver Instrument Co., Denver, USA). We recorded staff roles, commuting distances and transport modes, and measured task durations using stopwatches. We estimated electricity use (in kWh) based on manufacturer specifications and inputs from the biomedical engineer at the Medical Research Council Unit the Gambia, and apportioned per test by dividing total energy consumption by the average number of samples processed per use cycle (e.g. per centrifuge run). At Banjulinding, where on-site PCR was unavailable, we recorded sample referral details, that is distance travelled, mode of transport and sample volume. For patient return travel, we estimated average distances using residential data and Google Maps. We grouped transport modes as vans (14 seats), taxis (four seats) or walking. Based on interviews, most women used vans, about a fifth used taxis, and women living within 1 km of the health centre walked.

For stage 8 (end-of-life), we manually inspected and weighed all test-related waste, including packaging. However, we excluded waste from ancillary devices required to operate the PCR platform (e.g. laptop, desktop computer, barcode scanner and printer), as these items are typically repurposed or continue to be used beyond the lifespan of the PCR device itself. We recorded waste transport distances and disposal methods (autoclaving, shredding or incineration) based on facility-level waste data (October 2023–September 2024). We also recorded the travel of waste management staff.

We obtained emission factors, defined as coefficients expressing greenhouse gas emissions per unit of activity (g CO_2_e per unit), from Fujirebio, previous studies^23^ and the Base Carbone database, version 23.4, produced by the French Agency for Ecological Transition. When unavailable, we used proxies based on comparable materials or processes, following standard methods and expert input.

### Data quality assessment

Following the Greenhouse Gas Protocol,^20^ we assessed data quality for five dimensions: technological, temporal and geographical representativeness; completeness; and reliability. We rated each indicator qualitatively from very good to poor (online repository).^24^ We converted the ratings into quantitative uncertainty values, expressed as ± percentage ranges, using the Bilan Carbone® method,^25^ version 9 (online repository),^24^ to estimate uncertainty for each activity data point. For emission factors, we used uncertainty values as reported by the French Agency for Ecological Transition or previous studies; for HBcrAg rapid diagnostic test, the manufacturer provided uncertainty estimates.

### Data analysis

We used Excel (Microsoft, Redmond, USA) to compile activity data and emission factors for each product or process. We categorized emissions as test-dependent (consumables and test materials) or site-dependent (transport, use and waste management), to reflect whether they arise from the diagnostic product itself or from facility-level workflows and infrastructure (Table 1). We made the calculations in two steps. First, for each product or process within a functional unit, we estimated emissions by multiplying activity data by its emission factor. Uncertainty was calculated using:
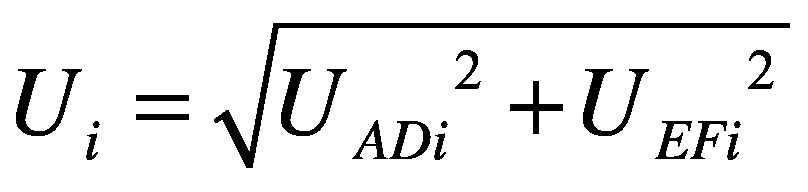
(1)where *U_i_* is the combined uncertainty (%) for the *i*th product or process, *U*_AD_*_i_* is the uncertainty (%) in activity data and *U*_EF_*_i_* is the uncertainty (%) in the emission factor.

**Table 1:** Carbon footprint of antenatal diagnostic strategies to identify pregnant women eligible for hepatitis B antiviral prophylaxis by health care facility, the Gambia, 2024

Domain and item	Carbon dioxide emitted, g															
	Bwiam (rural general hospital)				Banjulinding (suburban health centre)					Brikama (suburban district hospital)				Bundung (urban maternal and child hospital)		
	Rapid diagnostic test		Point-of-care PCR		Rapid diagnostic test			Point-of-care PCR		Rapid diagnostic test		Point-of-care PCR		Rapid diagnostic test		Point-of-care PCR
	Capillary blood	Plasma			Capillary blood		Plasma			Capillary blood	Plasma			Capillary blood	Plasma	
**Last-mile transportation**	18.9	24.1	44.7			1.9	2.5	4.5		5.8	7.4	13.7		1.9	2.4	4.4
**Consumables**																
Sampling equipment																
Gloves	40.0	40.0	40.0		40.0		40.0	40.0		40.0	NA	NA		40.0	NA	NA
Cotton	1.7	1.7	1.7		1.7		1.7	1.7		1.7	NA	NA		1.7	NA	NA
Alcohol	0.9	0.9	0.9		0.9		0.9	0.9		0.9	NA	NA		0.9	NA	NA
Syringe and needle	NA	9.5	9.5		NA		9.5	9.5		NA	NA	NA		NA	NA	NA
Finger pricker	7.8	NA	NA		7.8		NA	NA		7.8	NA	NA		7.8	NA	NA
Blood sample tube	NA	11.5	11.5		NA		11.5	11.5		NA	NA	NA		NA	NA	NA
Testing preparation																
Gloves	NA	40.0	40.0		NA		40.0	40.0		NA	40.0	40.0		NA	40.0	40.0
Transfer pipettes	NA	NA	3.6		NA		NA	3.6		NA	NA	3.6		NA	NA	3.6
Pipette tips	NA	0.7	NA		NA		0.7	NA		NA	0.7	NA		NA	0.7	NA
**Test**																
HBcrAg rapid diagnostic test	129.0	129.0	NA		129.0		129.0	NA		129.0	129.0	NA		129.0	129.0	NA
Point-of-care PCR (HBV viral load cartridge)	NA	NA	290.0		NA		NA	290.0		NA	NA	290.0		NA	NA	290.0
Point-of-care PCR (machine, module IV)	NA	NA	NA		NA		NA	18.6		NA	NA	NA		NA	NA	15.2
Point-of-care PCR (machine, module XVI)	NA	NA	31.6		NA		NA	NA		NA	NA	35.3		NA	NA	NA
Printer (device)	NA	NA	4.7		NA		NA	10.9		NA	NA	5.3		NA	NA	8.9
Desktop (device)	NA	NA	9.0		NA		NA	NA		NA	NA	10.1		NA	NA	17.2
Laptop (device)	NA	NA	NA		NA		NA	19.4		NA	NA	NA		NA	NA	NA
Barcode scanner (device)	NA	NA	0.3		NA		NA	0.7		NA	NA	0.3		NA	NA	0.6
**Product use**																
Energy for sample conservation before point-of-care PCR HBV viral load test is done	NA	NA	NA		NA		NA	9.6		NA	NA	NA		NA	28.7	28.7
Energy to run a sampling room	27.2	1.8	1.8		0.2		0.2	0.2		23.8	NA	NA		23.8	NA	NA
Transportation of the sample to the Medical Research Council Unit the Gambia	NA	NA	NA		NA		NA	5.7		NA	NA	NA		NA	NA	NA
Energy to run a laboratory	NA	151.1	912.2		101.5		135.8	842.2		NA	174.3	912.2		NA	174.3	872.4
Laboratory technician’s travel	21.9	26.7	9.7		21.9		26.7	9.7		37.6	29.2	NA		25.2	19.6	NA
Skilled laboratory technician’s travel	NA	NA	15.8		NA		NA	28.7		NA	NA	33.3		NA	NA	22.3
Women’s travel	NA	NA	NA		NA		NA	306.1		NA	NA	205.4		250.1	250.1	250.1
Midwives’ travel	12.1	12.1	12.1		39.1		39.1	39.1		47.7	47.7	47.7		48.7	48.7	48.7
**End-of-life and waste**																
Waste management staff travel	NA	NA	NA		1.5		1.5	1.5		1.5	1.2	1.2		1.5	1.2	1.2
Transportation of waste to management site and dumpsite	NA	NA	NA		7.3		6.3	20.6		6.5	1.8	3.4		8.8	4.3	8.4
Energy for waste management	21.9	39.0	62.1		6.8		8.6	13.0		6.8	4.6	9.0		7.8	4.6	9.0
Point-of-care PCR, machine end-of-life	NA	NA	4.9		NA		NA	2.9		NA	NA	5.6		NA	NA	2.4
**Total ± uncertainty**	**281.4 ± 34.8**	**488.1 ± 48.8**	**1506.1 ± 199.6**		**359.6 ± 40.9**		**454.0 ± 45.3**	**1730.6 ± 182.0**		**309.1 ± 37.5**	**435.9 ± 48.5**	**1616.1 ± 208.6**		**547.2 ± 88.3**	**703.6 ± 93.8**	**1623.1 ± 212.2**

HBcrAg: hepatitis B core-related antigen; HBV: hepatitis B virus; NA: not applicable; PCR: polymerase chain reaction.

Second, we obtained total carbon emissions per diagnostic strategy by summing all emissions from individual products and processes. We propagated overall uncertainty (%) from the individual uncertainties using the following formula:
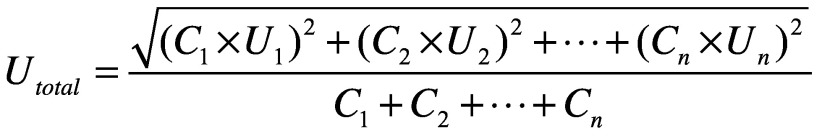
(2)where *U*_total_ is the total propagated uncertainty (%), *C*_i_ is the emission of the *i*th product or process and *U_i_* is its combined uncertainty (%).

We applied the *U*_total_ to the estimated CO_2_e and report the uncertainty as ± gram ranges. We adapted the propagation equations from the Greenhouse Gas Protocol which align with the Intergovernmental Panel on Climate Change method.^26^

We used the Wilcoxon rank-sum test for pairwise comparisons between diagnostic strategies. We performed two sensitivity analyses, one excluding air conditioning during testing with rapid diagnostic tests and the other modelling savings in emissions when test results were provided during the same visit as HBsAg screening.

## Results

### Testing workflows

**Fig. 3:**
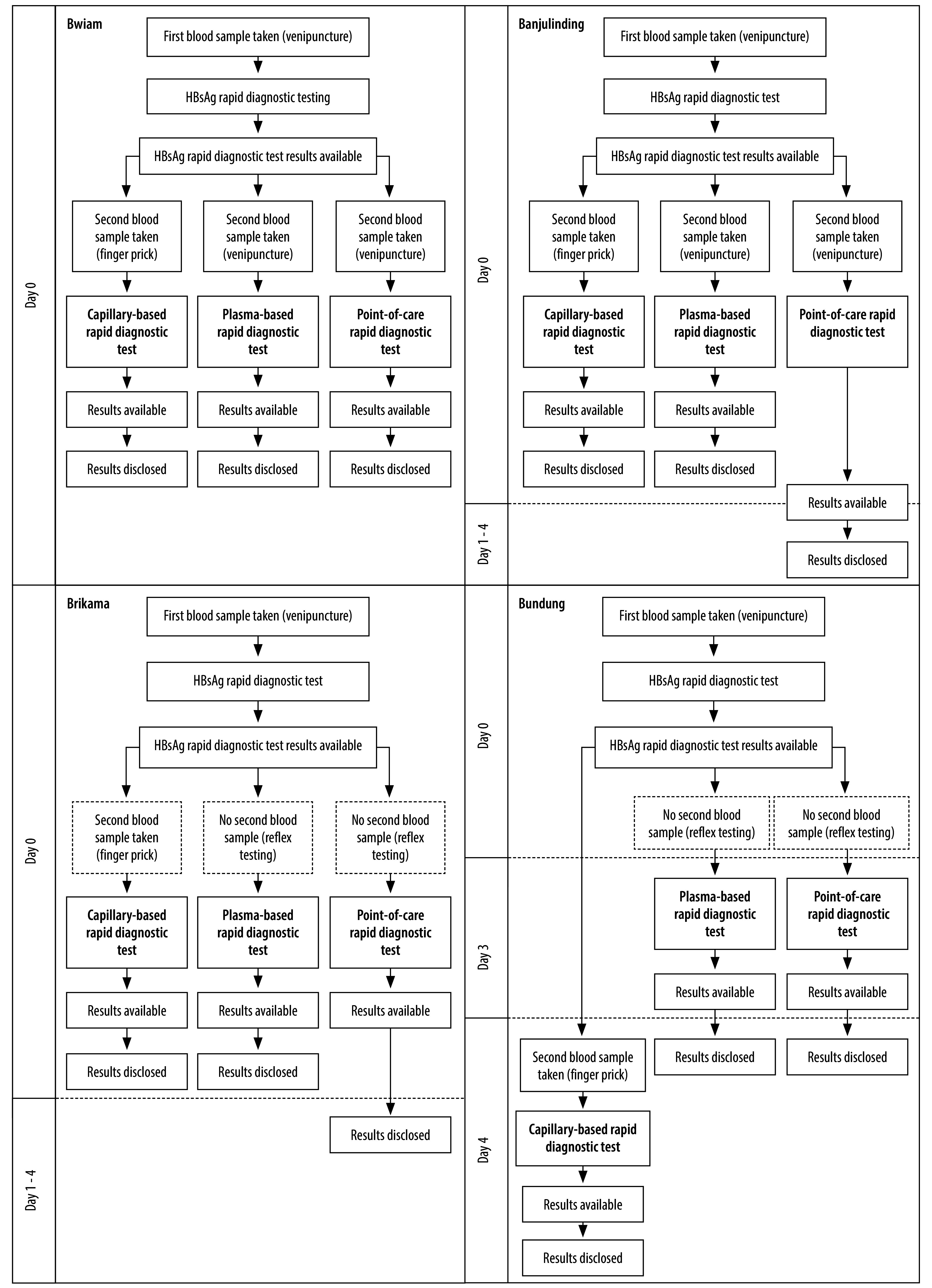
Workflows for identifying pregnant women eligible for hepatitis B antiviral prophylaxis in antenatal care, by health-care facility, the Gambia, 2024

HBsAg: hepatis B surface antigen; PCR: polymerase chain reaction.

All facilities conducted antenatal screening on fixed days, beginning with venous blood collection for multiple tests, including HBsAg (Determine 2, Abbott, Illinois, USA). Care pathways following sample collection varied in the four sites (Box 1; Fig. 3 and online repository).^24^

Box 1 **Testing workflows for health facilities included in the study on carbon emissions associated with antenatal testing for hepatitis B prophylaxis eligibility, the Gambia, 2024**

***Bundung (urban maternal and child health hospital)***

Screening was done on Mondays and all women were asked to return on Friday for their results. For women who were HBsAg-positive, reflex testing using the same plasma sample was performed during the week with both point-of-care PCR and plasma rapid diagnostic test. The capillary rapid diagnostic test required a finger prick on Friday. Results for both screening and post-screening tests were provided on Friday.

***Brikama (suburban district hospital)***

Women waited on-site for HBsAg results. If positive, reflex testing used the same plasma for point-of-care PCR and plasma rapid diagnostic test; the capillary rapid diagnostic test required a separate finger prick. Rapid diagnostic test results were available on the same day, whereas PCR results were delayed by 1–4 days, requiring a return visit.

***Bwiam (rural general hospital) and Banjulinding (suburban health centre)***

Reflex testing was not done. Instead, a second venous sample was collected after a positive HBsAg result. In Bwiam, all tests were completed on the same day and results were delivered during the same visit. At Banjulinding, the rapid diagnostic test was performed on-site, but PCR samples were sent to the laboratory of the Medical Research Council Unit the Gambia, requiring a return visit for results.

### Waste stream

We documented the full waste stream at each site and identified two main management pathways: on-site and off-site processing. At Bwiam, all waste was incinerated on-site using the INCINER8 I8-M70 high-temperature incinerator (Inciner8 Limited, Burscough, England), at 850–1200 °C. At the other facilities, waste was segregated and transported to the centralized clinical waste treatment centre in Farato (Fig. 1). At this centre, infectious waste, accounting for 72.4% (29 442/40 685 kg) of total waste generated between October 2023 and September 2024, was autoclaved and shredded using the Ecosteryl 250 system (Ecosteryl, Mons, Belgium), designed to reduce waste by 80%; residual waste was sent to the Brikama dumpsite. Sharps waste, representing 27.6% (11 243/40 685 kg) of total waste, was transferred to the National Public Health Laboratory for incineration using the INCINER8 I8-M70 system.

### Carbon footprint

**Fig. 4:**
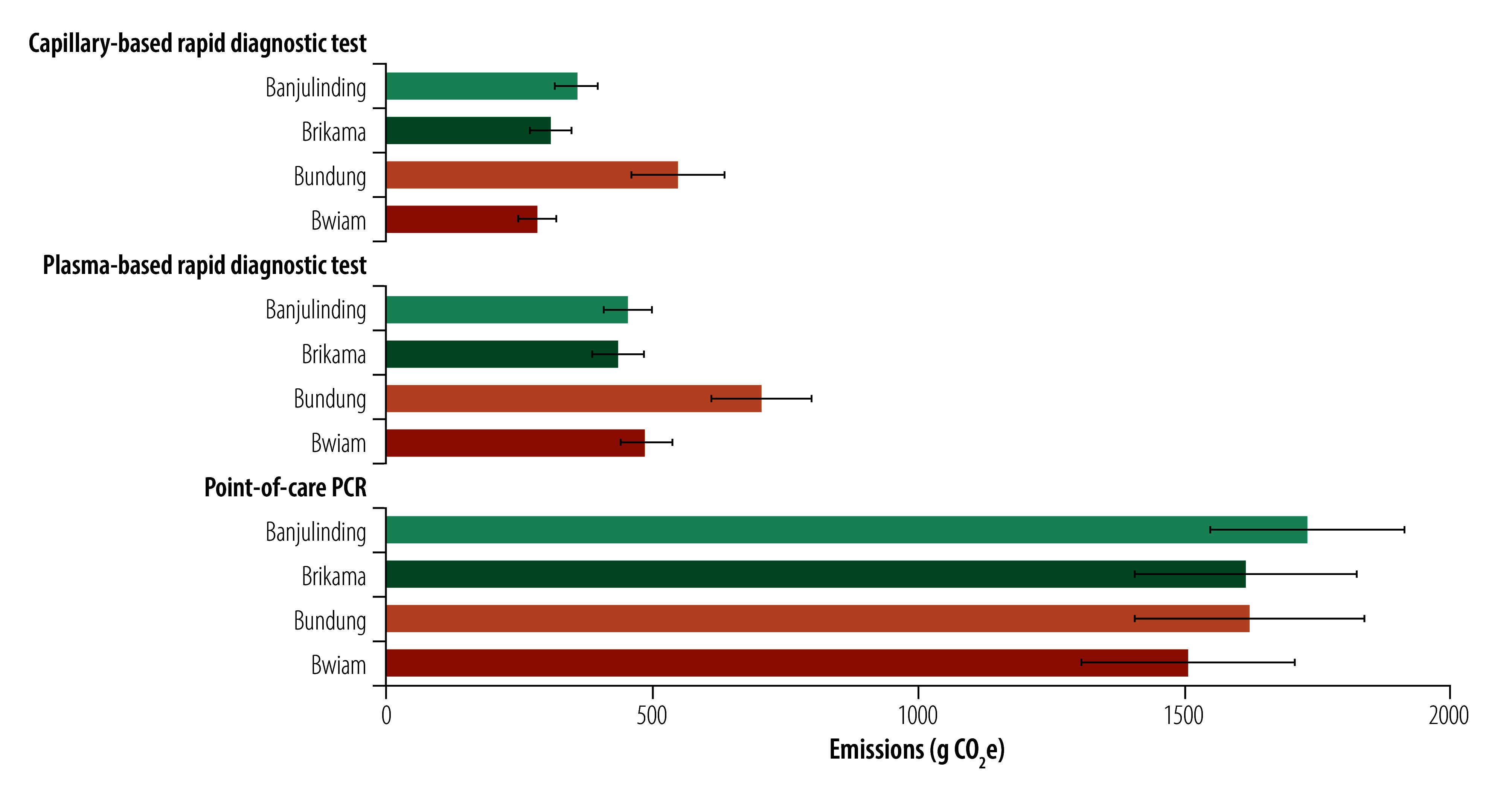
Carbon footprint of hepatitis B antenatal diagnostic strategies in each health-care facility, the Gambia, 2024

At all sites, the carbon footprint per testing visit followed a consistent pattern: highest for the point-of-care PCR strategy, intermediate for the plasma rapid diagnostic test and lowest for the capillary rapid diagnostic test (Fig. 4). Average emissions were 1619.0 ± 200.6 g CO_2_e for point-of-care PCR, 520.4 ± 59.1 g CO_2_e for plasma rapid diagnostic test and 374.3 ± 50.4 g CO_2_e for capillary rapid diagnostic test. Pairwise comparisons using the Wilcoxon rank-sum test showed significantly lower emissions for capillary rapid diagnostic test compared with PCR (*P*-value: 0.028), and for plasma rapid diagnostic test compared with PCR (*P*-value: 0.028), while the difference between capillary and plasma rapid diagnostic test was not statistically significant (*P*-value: 0.200).

CO_2_e: carbon dioxide equivalent; PCR: polymerase chain reaction.

Note: the error bars indicate uncertainties, calculated as described in the methods section.

**Fig. 5:**
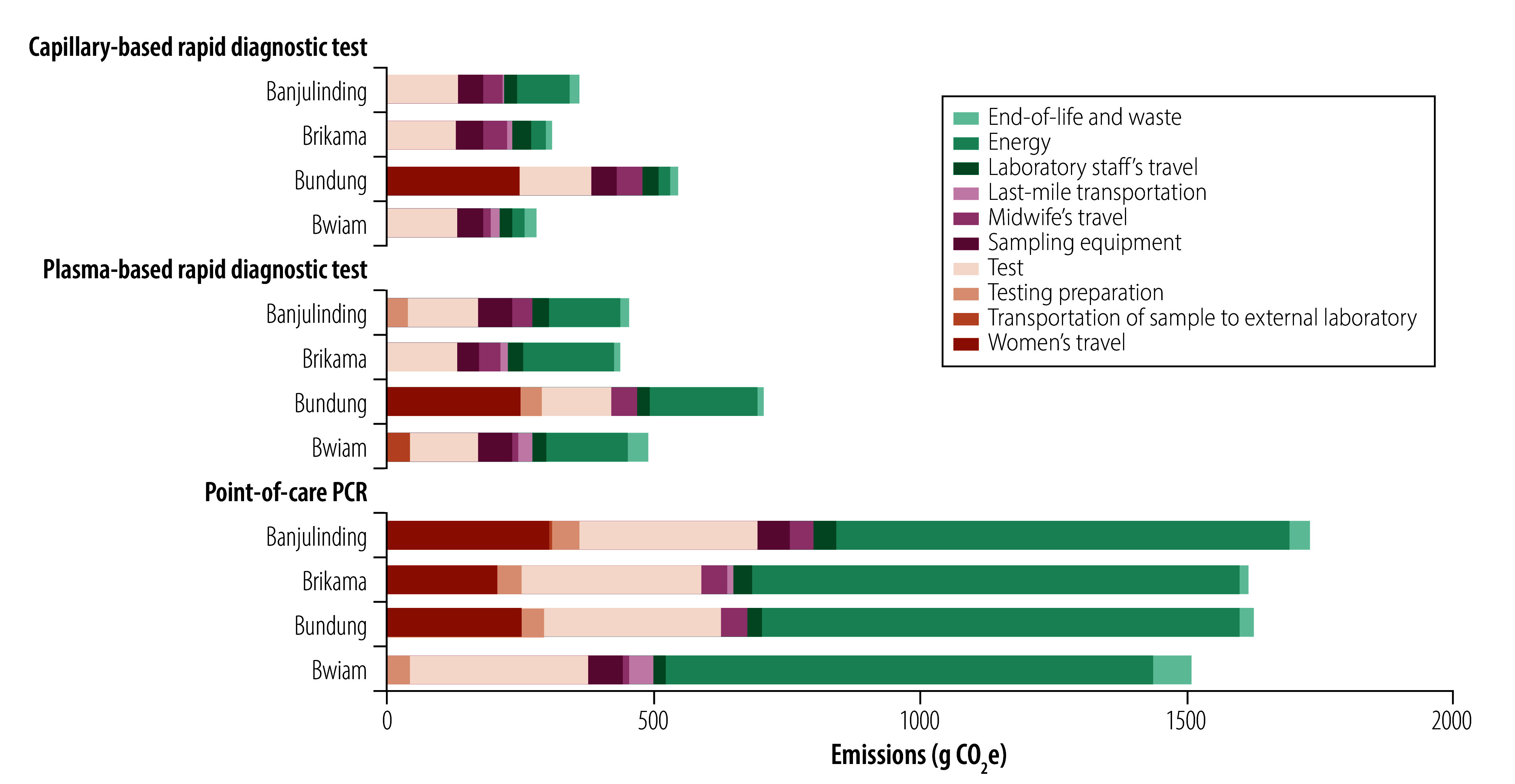
Source of carbon emissions of hepatitis B antenatal diagnostic strategies in each health-care facility, the Gambia, 2024

Emission sources varied by strategy (Table 1, Fig. 5 and online repository).^24^ For point-of-care PCR, the main contributor was energy use (54.1%; 894.8/1655.1), mainly from air conditioning (84.8%; 759.1/894.8 of energy-related emissions), followed by the test cartridge and device (20.4%; 336.9/1655.1) and women’s travel (11.5%; 190.4/1655.1). For plasma rapid diagnostic test, emissions came mainly from energy use (30.6%; 166.6/544.6), the test itself (23.7%; 129.0/544.6) and consumables (17.7%; 96.6/544.6). For capillary rapid diagnostic test, key contributors were the test (34.5%; 129.0/374.3), women’s travel (16.7%; 62.5/374.3), consumables (13.4%; 50.3/374.3), energy use (11.8%; 44.1/374.3) and laboratory staff travel (7.1%; 26.6/374.3).

CO_2_e: carbon dioxide equivalent; PCR: polymerase chain reaction.

In all four sites, point-of-care PCR produced three to four times more emissions than rapid diagnostic testing strategies, mainly due to its reliance on air conditioning (759.1 g CO_2_e for point-of-care PCR versus 125.2 g CO_2_e for plasma rapid diagnostic test, and 24.3 g CO_2_e for capillary rapid diagnostic test); the test itself (290.0 g CO_2_e for point-of-care PCR versus 129.0 g CO_2_e for rapid diagnostic test); and PCR-specific requirements such as the PCR device (47.0 g CO_2_e) and additional travel by women to collect viral load results in Banjulinding and Brikama (255.8 g CO_2_e, averaged across the four sites).

The higher footprint of plasma rapid diagnostic test over capillary rapid diagnostic test was mainly due to extra consumables in testing preparation (gloves and transfer pipettes: 40.7 g CO_2_e) and greater energy use for plasma separation (15.9 g CO_2_e).

### Carbon footprint by site

Carbon emissions showed less variation between sites using the same diagnostic strategy (Fig. 4). For point-of-care PCR, Bwiam had the lowest emissions, as testing occurred on the same day as HBsAg screening, avoiding extra travel for women. However, emissions were higher for last-mile transport, due to Bwiam’s distance from the National Public Health Laboratory (Fig. 1) and from on-site incineration using the INCINER8 I8-M70 system.

For both rapid diagnostic test strategies, Bundung had the highest emissions, as it was the only site where women returned on a separate day to receive both screening and post-screening test results. Banjulinding had the second highest emissions for capillary rapid diagnostic test, mainly due to the use of an air-conditioned laboratory, which increased energy-related emissions.

### Sensitivity analysis

**Fig. 6:**
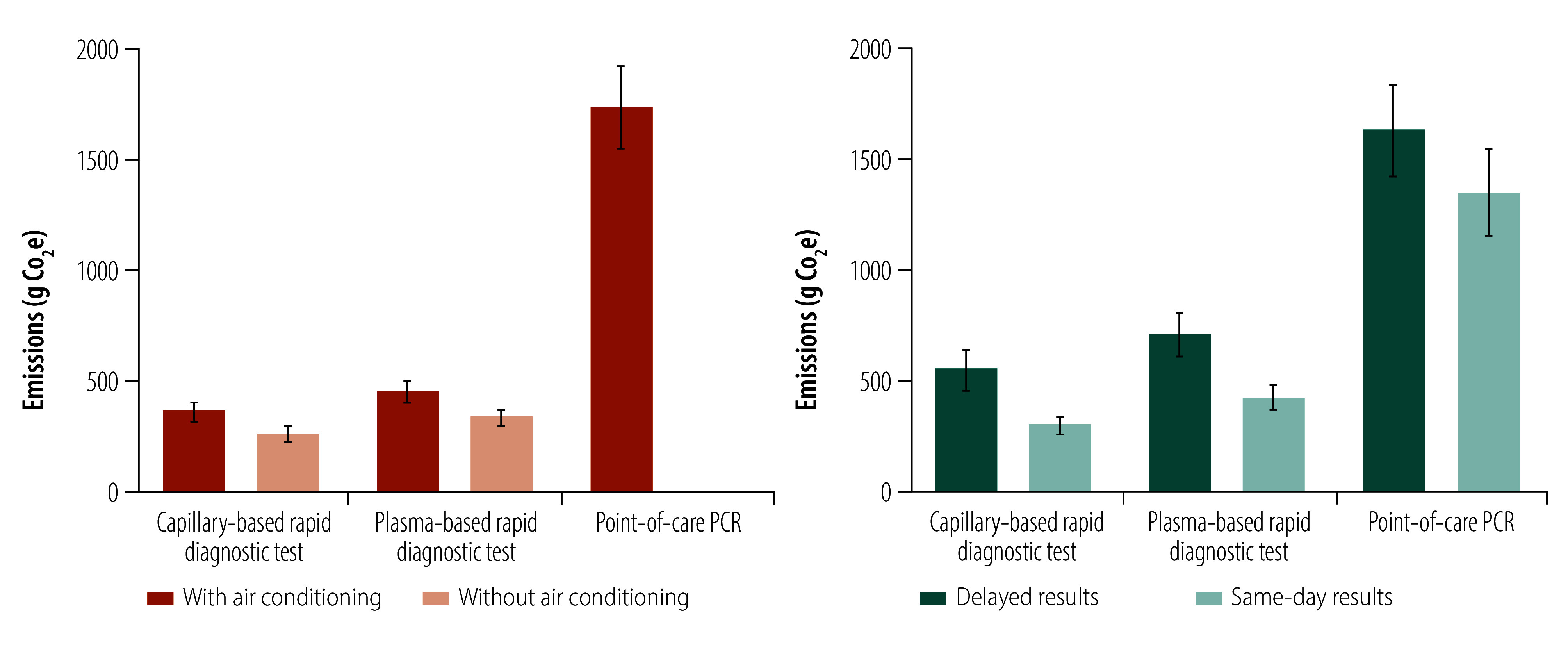
Total carbon footprint by diagnostic strategy, with and without air conditioning, and time to results at Bundung maternal and child health hospital, the Gambia, 2024

Since the HBcrAg rapid diagnostic test can be performed at room temperature, we modelled a scenario in Banjulinding excluding air conditioning. This model reduced the carbon footprint from 359.6 g to 262.3 g CO_2_e for the capillary rapid diagnostic test, and from 454.0 g CO_2_e to 335.4 g CO_2_e for plasma rapid diagnostic test (Fig. 6). This model was not feasible for point-of-care PCR, which requires ambient temperatures lower than 30 °C for accurate performance.

CO_2_e: carbon dioxide equivalent; PCR: polymerase chain reaction.

Note: the error bars indicate uncertainties, calculated as described in the methods section.

In Bundung, where women routinely returned to collect results, we estimated emissions savings if performing the test and providing the results occurred on the same day as HBsAg screening. This model would reduce emissions by 250.1 g CO_2_e for capillary rapid diagnostic test, 278.8 g CO_2_e for plasma rapid diagnostic test and 250.1 g CO_2_e for point-of-care PCR (Fig. 6).

## Discussion

This study adds to the limited evidence on the carbon footprint of diagnostic strategies in a resource-constrained setting. Using a life cycle assessment, we found that the point-of-care PCR test generated the highest emissions per test, followed by the plasma-based rapid diagnostic test, with the capillary-based rapid diagnostic test having the lowest footprint. The elevated emissions from point-of-care PCR were mainly driven by its high energy requirements, particularly air conditioning to maintain laboratory temperatures lower than 30 °C, which is critical for optimal functioning of the point-of-care PCR platform.^27^ Additional contributors included emissions from the test cartridge and device, as well as additional travel by women returning to collect point-of-care PCR results in facilities where same-day reporting was not available.

Few studies have assessed the environmental impact of diagnostics for the same clinical indication. Research in the United Kingdom of Great Britain and Northern Ireland comparing cervical cancer screening methods found self-sampling had lower emissions than clinician-based methods.^14^ In France, histology and immunohistochemistry were assessed to identify high-impact steps in pathology workflows.^15^ In the USA, prostate biopsy pathways incorporating prostate magnetic resonance imaging had substantially higher greenhouse gas emissions than ultrasound-guided biopsy alone due to the energy-intensive nature of magnetic resonance imaging.^28^ While these studies provide important examples of integrating environmental considerations into health technology assessment, all were conducted in high-income countries. Our study fills a gap by demonstrating that life cycle assessments are feasible in low- and middle-income countries and that simplified diagnostics, such as the HBcrAg rapid diagnostic test, may be both environmentally efficient and context-appropriate.

Capillary rapid diagnostic tests may reduce emissions by over 75.0% compared with point-of-care PCR, potentially saving about 1.25 kg CO_2_e per test. Applying this saving to the Gambia’s antenatal programme, which serves an estimated 3700 HBsAg-positive women annually,^29^^,^^30^ translates to an annual reduction of 4600 kg CO_2_e, equivalent to about 18 000 km driven by a mid-sized car. These savings could scale up substantially if adopted across other HBV-endemic countries. Sensitivity analyses indicated that simple adaptations for rapid diagnostic test strategies, such as removing air conditioning or delivering results on the same day, could further reduce emissions by 20.0–30.0%.

Importantly, the rapid diagnostic test strategy not only has the lowest emissions but also offers the most resilient and resource-efficient option. Unlike point-of-care PCR, rapid diagnostic testing does not rely on air conditioning or specialized equipment, making it less vulnerable to power outages or system failures, risks that are increasing with climate change. By minimizing technological dependencies, the rapid diagnostic test method aligns sustainability with operational resilience, supporting continuity of care even during blackouts or heatwaves. Choosing simpler, low-tech options can thus simultaneously advance climate and health-system goals in resource-constrained settings. 

Our study has some limitations. Emission factors for upstream processes (i.e. processes occurring before on-site test use) relied on secondary sources or proxies, although we aligned with international standards. Our functional unit was a single antenatal testing episode; we did not assess diagnostic accuracy or downstream impact of missed diagnoses on health-care use or emissions. These considerations were beyond the scope of this analysis and will be explored in future work. Lastly, our study was not designed to generate nationally representative estimates, and our findings may not be generalizable to settings with different health-care infrastructure, energy sources or waste management systems.

In summary, our study shows that diagnostic strategies for hepatitis B may differ markedly in their environmental impact. Rapid diagnostic test using capillary blood may offer a lower-carbon alternative to point-of-care PCR-based testing, with further reductions possible through context-appropriate workflow adjustments. Incorporating environmental metrics into health technology assessments could support more sustainable and equitable approaches to infectious disease control in low- and middle-income countries.
